# Hospital Meetings

**Published:** 1902-03-08

**Authors:** 


### The Institutional Workshop.

### HOSPITAL MEETINGS.

### THE ROYAL FREE HOSPITAL.

A RECORD ANNUAL MEETING.

The meeting on Wednesday afternoon last week was certainly as unlike an ordinary hospital meeting as it could well be. "Dull" is a word that may fitly be used to describe many of these functions, and the exception to the rule only proves that dulness is not an essential of business. For nothing but routine business was transacted at the Royal Free annual meeting, and yet it was made interesting. There were plants on the platform, flowers on the table, ladies on the platform and in the audience, flags were festooned across the ceiling of the Calthorpe Ward, where the meeting took place, and last, but not least, a lady was in the chair. Not that H.R.H. Princess Christian discharged the active duties of a chairman; these were delegated to Mr. Charles Burt, chairman of the weekly board, but her presence gave great pleasure, and she seemed to enjoy the position. Before the meeting began, the Princess was conducted through the wards by the senior physician, the senior surgeon, Mr. Charles Burt, and Miss Wedgewood, the matron. When she arrived at the meeting, Miss Naylor, M.B., London, presented her, on behalf of the students, with a beautiful bouquet of lilies of the valley and pale lilac. There were on the platform the Princess, Mr. Burt, Mrs. Garrett Anderson, M.D., Captain Jessel, and Sir Edwin Durning-Lawrence. Among the audience were Mrs. Scharlieb, M.D., the medical staff of the hospital (men and women), the students, and a few of the nurses.

Letters of regret had been received from the Earl of Stamford, Lord Rowton, Lord Leigh, and Mr. Randolph Churchill, who were unable to be present, and after the notice convening the meeting had been read, Mr. Burt made a statement of the condition of the hospital, addressing his remarks to the Princess. The annual meeting, he said, was the occasion on which those who had been managing the hospital for a year had to give an account of their stewardship, and be re-elected if they had done well, and turned out if they had not. He hoped the meeting would not cashier the managers until they had heard what work they had done.

The Position of the Hospital.

The number of patients treated during the year, said Mr. Burt, was very large; of 2,140 in-patients (110 more than in 1900), 1,774 were either cured or relieved; and there were 37,305 out-patients and casualties, a large number of the latter being victims of cab accidents. Since the hospital was founded, in February 1828, no fewer than 2½ millions of persons had been treated, a right royal number, he thought. How were these patients paid for? The cost of maintenance had increased, and putting down each in-patient at £1 10s., the total cost of £13,099 during the year was nearly accounted for. Besides this amount, £4,300 had been invested—he used the word because the committee felt the improvements yielded a very good return—in additions and improvements. Those who knew what a nurse's work was could appreciate the fact that they now had each a little room in which to rest and be thankful.

From Hand to Mouth.

The senior resident medical officer, Mr. Burt continued, was the only one of the medical staff who received a salary, and his was only a modest fee. The other officers gave their time and services freely, and had their satisfaction in the work done. The matron gave herself, her life, her work, indeed everything, to the hospital, which lived on from one day to another, not knowing where its next day's meals would come from. He looked upon legacies as miracles, but unfortunately the legacies had had to be used to pay off tradesmen's debts, which, from being £1,300 last year, were now reduced to £430. The festival dinner realised £4,518, but it meant very hard work, and could not be done every year. The committee were very thankful to King Edward's Fund for giving them £750 for income, and £750 for the buildings. They had also given what they had given to nobody else, namely, a certificate "*that the hospital is deserving, and requires more support from the public.*" Two sums of £1,000 each had been given for the endowment of beds, and he preferred this form of gift to any other—for one thing there was no legacy duty to pay. The help given by Mrs. Garrett Anderson, who had collected from past and present students a sum sufficient to endow three beds for three years, was very greatly appreciated.

No Strike.

Mr. Burt's next remark called forth a good deal of applause. During last year, he said, two medical women were appointed as resident medical officers, and the Royal Free was the first, certainly of London general hospitals, to do this. In another place such an appointment had led to a strike, but at the Royal Free the committee had the medical staff with them, and the arrangement was working exceedingly well. They trusted this arrangement would go on as long as the hospital continued. Much as had been done to improve the hospital, it must not be supposed that the work was finished. To keep pace with science, improvements were constantly needed. The two things now most urgently wanted were electric lifts and new kitchen apparatus, the latter having been in use for 40 years. He concluded by hoping that the Princess would occupy the chair at the next annual meeting.

Mr. Gamlen, who said he was one of the 2½ million who had received benefit from the hospital, seconded the adoption of the report, and it was accepted by the meeting.

The Late President.

A resolution of sympathy with Lady Dufferin and her family upon the death of the Marquis of Dufferin and Ava, who had been President since 1889, was moved by Captain Jessel and seconded by Mrs. Garrett Anderson, who spoke of the beneficent work of Lady Dufferin in India, and its effect on the openings for medical women there.

The New President.

That H.R.H. Princess Christian of Schleswig-Holstein be appointed President of the Hospital, was moved by Sir Edwin Durning Lawrence and seconded by Mrs. Scharlieb, and the Princess requested Mr. Burt to say that she was very pleased to accept the position.

Other resolutions referred to the re-appointment of officers and thanks to all connected with the hospital, and among the speakers were Mr. F. D. Mocatta and the Vicar of Clerkenwell.

At the close of the meeting tea was provided in the lecture room.

### KING'S COLLEGE HOSPITAL.

IS DEBT A GOOD THING?

The only interesting question raised at the annual meeting of King's College Hospital on Thursday last week was that put by one of the oldest governors, Mr. S. J. Wilde. The report, which had been circulated, and was therefore taken as read, was before the meeting, which consisted practically of the Committee of Management, although a personal notice had been sent to all the 900 governors, and an advertisement had been inserted in the press.

"Does the hospital do right," Mr. Wilde asked, "to go beyond its income?" He went on to speak of the hospital for accidents at Poplar, and said that the first thing that attracted him to it was that it was never in debt, and that it had thriven upon this fact. It never spent any money until it had it. There were other attractive features about it, one of which was the enormous interest taken in its welfare by the poor people of the neighbourhood, who took collecting boxes, etc. That, he supposed, would be an impracticable way of raising money for King's, but he returned once more to the question whether it was a good thing to spend more than you got? Could not the expenditure be brought a little more within the limits of income, even if it meant taking fewer patients?

To this question the Rev. Dr. Robertson, Principal of King's College, was eager to reply. He moved the adoption of the report. He said nobody could be more anxious than the late committee had been, or than the new one, he doubted not, would be, to keep the expenditure as far as possible within the limits of income. If Mr. Wilde would carry his mind back some few years, say ten or twelve, he would see that very great progress had been made towards reducing the debt, and that without limiting the number of patients. He himself had been a member of the committee for five years, and during that time he did not think a single penny had been wasted, nor did he think anyone would wish them to resort to the extreme measure of closing the wards. It happened that just when the accounts had to be made up, they were in a less satisfactory relation to the bank than at other times. They were always anxious to cut their coat according to the cloth, but the cry in coming years would be for more cloth.

Lieut.-Col. Fellowes, in seconding the adoption of the report, said he could not claim to have been very long a governor—only some sixteen years—but was it quite accurate to say that this charity had always been in debt?

The Chairman, Mr. Charles Awdrey (treasurer), said there were certain difficulties in acting, as they would like to do, upon Mr. Wilde's suggestion. The subscriptions and donations did not amount to more than a quarter of what had to be spent. It was practically impossible not to be in debt, though they would like to get as near as possible to an equality between income and outlay. It must be remembered, however, that if there was no debt, they might lose friends; he knew there were some who, when they got a report showing a balance at the bank, thought there was no necessity to help.

The report was adopted unanimously, and the rest of the meeting consisted of a succession of resolutions, mainly of thanks to officers of the hospital, including the sister-matron, sisters and nurses, for their devoted services; it was largely through them that the patients were cured in a shorter time than used to be the case. The hospital prided itself on its nursing staff, and that they were able to do so was largely due to the sister-matron, who gave the tone to those working under her. A bye-law, practically, as it was explained, "making it impossible to hold a meeting in August, a month when no meeting was ever held," was also proposed, seconded, and carried.

---

### THE MIDDLESEX HOSPITAL.

### ANNUAL MEETING.

This was presided over by Lord Sandhurst, G.C.S.I., and about 40 governors were present, including Sir Ralph Thompson, G.C.B., Sir Arthur Townley Watson, Colonel Gathorne Hardy, Colonel Charles Needham, Mr. G. I. Marjoribanks, the Hon. G. W. Spencer Lyttelton, C.B., and the matron, Miss Thorold. The meeting, which was held at noon on Thursday last week, began with prayer, after which the secretary read the notice convening the meeting, which had appeared in the principal daily papers, and had been posted in the board-room. The entire report of 16 pages was then read by the secretary, and its adoption was moved by Sir Ralph Thompson and seconded by Sir Arthur Watson. While it was being read the treasurer collected the alms-boxes and counted their contents, which were found to amount to £87 7s. 5d., or £12 5s. 5d. more than last year.

Sir Ralph Thompson said the hospital could not cut its coat according to its cloth, for it had to spend money whether it had it or not. The generous public had always helped the funds, and he thought that if they were to act in any other way than that to which they were accustomed, they would be landed in an awkward position. A large expenditure only showed healthy progress, and the laundry and nurses' quarters alluded to in the report were both necessary. With regard to the investigation of cancer, it was satisfactory to know that the Middlesex Hospital had given an impetus to the subject which had "caught on" with the public. They must go on, in this department also, in faith. Dr. Caley, who had retired in consequence of the age limit in the laws of the hospital, would be very much missed; as also would Dr. Arthur Robinson, who had been elected to the Chair of Anatomy at King's College. The revision of laws had been a work of considerable labour, and they were greatly indebted to the special committee who had undertaken it. As to the medical school, the numbers had fallen short of what he had hoped to see, but the Middlesex was not singular in this "shrinkage," to use a business term; it was, he believed, much the same in all the London schools. No mention was made in the report of the admirable way in which the hospital had been served by the permanent staff; it meant considerable financial sacrifice as well as time, and they had only the reward of the old proverb. No hospital was better served by its permanent staff than the Middlesex.

The report having been adopted and some other business details transacted, Mr. Hoare expressed his pleasure at being made a vice-president. He had retired from the office of treasurer, he said, because he felt that a treasurer should be always at hand, and he was taking up his permanent residence in the country. He was very glad that his friend Mr. Burnand was to take his place. Dr. Caley was elected member of the medical committee, and then the general court resolved itself into a special court to consider the alteration of rules. These were made 21 years ago, and a special committee to consider them was appointed in 1897, consisting of Sir Arthur Watson, Mr. W. E. Gillett, and Lieut.-Col. T. Lambert Mears.

Sir Arthur Watson said that there was only one alteration which he thought it necessary to bring before the court, and that related to the radius within which it was thought advisable that the physicians and surgeons should live. The special committee thought that three miles was a fair limit. This would not apply to Mr. Andrew Clarke, in whose case he recommended that the clause should be suspended, the appointment of an assistant rendering it unnecessary in his case. Neither would it apply to such appointments as that of dental surgeon, etc. The alteration was adopted, and a vote of thanks to Lord Sandhurst closed the meeting, in which no incident of special interest took place.

---

### THE GREAT NORTHERN CENTRAL HOSPITAL.

The number of lady governors present was a noticeable feature of the annual meeting of the Great Northern Central Hospital on Friday afternoon last week. There are some 1,500 subscribers and donors to this hospital, and to each one an invitation is sent, hence the very good attendance both of men and women on Friday.

As might be expected, the question of funds occupied the meeting, to the exclusion of almost every other matter. There is a debt of £10,200 to the bankers, and some £600 will have to be spent on drainage, which it was believed was in an eminently satisfactory condition. The epidemic among the staff at the beginning of the year having caused some anxiety, the chairman, Sir John Dickson-Poynder, personally investigated the matter, and the London Sanitary Association kindly undertook to make an examination free of charge. Mr. Anderson, their engineer, found that serious defects existed, some of which were due to the design of the period at which the drains were constructed, and others to uncovered joints and leakages apparently consequent on slight settlements which have occurred in various parts of the building since its erection in 1889. The recommendations of Mr. Anderson are to be carried out, and will, it is believed, put the hospital in a thoroughly sound sanitary condition.

The financial condition is far less sound, and great expectations are based upon the Festival Dinner, to be held on May 12th, when the Duke of Marlborough will take the chair at the Hôtel Métropole. To keep the institution from debt, £8,000 annually is required, over and above the income which may be regarded as reasonably secure. During the last three years the receipts have failed to balance the expenditure. Although the legacies were higher last year than in 1900, part had to be invested for endowment; and higher prices of commodities, annual cleaning, repairs, interest on loan, and increase of the salaries list, necessitated by additions to the nursing staff, account for the increase of expenditure, which is £453 6s. 3d. more than in 1900. These were the points dealt with by Sir J. Dickson-Poynder in his opening remarks. The suggestion of Mr. Blackney, that a systematic house-to-house canvas should be undertaken with a view to raising small yearly subscriptions in the neighbourhoods of Muswell Hill, Hornsey, Finchley, etc., was warmly received, and the chairman promised that it should have the careful attention of the Management Committee.

The Hon. R. A. Capel and Mr. W. Burdett-Coutts were re-elected hon. treasurers; and Mr. Ernest Newton was elected an honorary life governor, on account of the subscription of £31 10s. from the Highbury Quadrant Young Men's Society, whose nominee he was.

### DREADNOUGHT—THE SEAMEN'S HOSPITAL SOCIETY.

The annual Court of Governors of this hospital was held at the Institute of Chartered Accountants on Friday, last week. Mr. P. A. Nairne, one of the trustees, was in the chair; the German Consul-General represented H.I.M. the Emperor of Germany, who is a governor, and Mr. F. A. Baker attended in his capacity of solicitor to the society.

Mr. Percival Nairne, chairman, said that financially the year just closed had not been a successful one. The amount received in subscriptions, donations, and other voluntary contributions had equalled, and even slightly surpassed, that of former years, but the help from legacies had been very small. If they had to contemplate a corresponding expenditure in years to come, the outlook was a very serious one.

### The Condition of the Dreadnought.

A year ago a letter was received from the Prince of Wales's Hospital Fund, saying that the council considered the Dreadnought Hospital building wholly unsuitable for the work which the society is trying to do there, and that in the view of the council the whole of the building, with the exception of the Somerset ward, ought to be pulled down, and a modern hospital built. The suggestion was that the Lords of the Admiralty should be approached, the committee being practically their tenants, with a view to getting the hospital rebuilt.

This year Mr. Nairne announced that the Lords of the Admiralty had been approached without success. There seemed no likelihood of their consenting to undertake the work, and, being tenants only, the committee did not feel justified in doing it themselves. It had been pointed out that the building was obsolete and unsuited for the purpose, but the committee did not agree in this; they considered it to be still a good hospital, though not on the most modern system. The wards, for instance, were small; but the seamen looked upon this as one feature of comfort, and having regard to the fact that under an Act of Parliament it was not wholly a hospital, but also a convalescent home, where the sailors might stay on after recovery, it seemed reasonable to take this also into account when considering the question. Personally, he thought sanitary science in such a condition that the present was not a good time to begin building.

### Convalescent Home Refused.

It was a matter of regret, the Chairman went on, not to be able to make use of the offer of a convalescent home in the Isle of Wight, which would accommodate 40 patients. The offer was made through the Prince of Wales's Fund, and included the interest on a sum of £10,000 towards the maintenance of the home. In the present financial condition of the society the committee did not feel justified in accepting the offer, which would have landed them in an estimated annual expenditure of £1,300 beyond the endowment. They very much regretted not being able to see their way to accepting it. The opportunity, however, was one which benevolent people might well take advantage of, and it would, if endowed, be a very valuable addition to the society's system.

Sir Lawrence Herbert, G.C.B., in moving the adoption of the report for 1901, spoke of Sir Francis Lovell's mission on behalf of the School of Tropical Medicine, and said that Sir Francis had already attained great success in collecting subscriptions.

Admiral Sir Richard Tracey, K.C.B., in seconding, said he had never seen a hospital where the convalescent patients were so well attended to as at the Dreadnought; the patients themselves thought it perfect. They must hope that there was no need to consider the question of rebuilding seriously. The funds would not run to it. As a constant visitor, he was quite satisfied with it.

Sir Henry Burdett, K.C.B., vice-president, said it seemed to him that in view of the courage which had been displayed by the administrators of this hospital, and the large view they had taken of their duties, and their public spirit in having thrown themselves into the breach when other large hospitals in London declined to take the field, they deserved a better fate than a deficit of £7,516 18s. 4d.

### A National Hospital.

As one having intimate knowledge of all that had occurred in connection with the Colonial Office and the Seamen's Hospital, he knew that where others drew back, this hospital stepped in. Why was it? Because the one note always struck by the managers was national. Having done this, and prospered, when the suggestion came that the nation needed a school of tropical medicine, although it was a bold policy, it was the only policy open to them, to incur the expense they did. And what were the results? A revolution had been brought about by the increased knowledge of tropical disease, and this had given a value to our possessions in the colonies which, owing to climatic conditions, they lacked before.

There was now positive evidence that swamps might be made tolerable. Further, through the munificence of Mr. Craggs, yellow fever was being practically dealt with, and he believed that the world would eventually be freed from it, as England had been freed from the plague. If "Yellow Jack" were triumphed over a deficit of £7,516 ought not to be possible. It would be the grandest thing the society had ever done, and the praise of it should go forth, in public meetings and through the press and in every possible way. This would bring about a greater appreciation of the work of the society and should materially affect the finances.

Travelling Scholarships.

Mr. Craggs had given £300 a year for a travelling scholarship to promote the study of disease in the most unhealthy parts of the world. The results should make other people consider whether any object could yield a better return than the endowment of such travelling scholarships. Three more such scholarships, he understood, would be most valuable to the school.

The Re-building of the Dreadnought.

Mr. Nairne had spoken of sanitary science; he had said he did not think the time was ripe for erecting a new hospital. The speaker ventured to think the remark was made in some misapprehension. A hundred years ago hospital buildings were a material factor in the treatment of disease; to-day they had very little to do with it. Lord Lister's discovery proved that ordinary cleanliness and care were the main things, and that the buildings mattered comparatively little. As far as buildings went, time made little difference one way or another; but they must be looked upon much in the same way as an ordinary house, and their fitness for the particular work they had to do was the point to be considered. The building in question was practically a Government building, and on an old foundation. The trustees were not disposed to spend money on re-building if they could help it. They were in the position of tenants. The building was put up for the treatment of disease, and some structural alterations were necessary. It was essential that the owners should take care of their building, and spend a sum of money upon its up-keep. Old structures had to be renewed; walls and flooring became defective, and these were matters that affected the absolute owners, upon whom the responsibility rested. It was not a matter for the tenants, whose responsibilities ended with keeping the building in ordinary repair. When it came to a matter of spending £50,000, it ought to be represented very strongly to the landlords. If they were unwilling, it was absolutely necessary to take steps to induce them to do what other trustees had had to do. The sum of 1½ millions had been spent in the last 10 years on bringing up the London hospitals to modern requirements; £300,000 would have to be spent by two others. Outside London, 1¾ millions had been spent in the last ten years in similar work. These were facts that ought to be brought to the notice of the trustees of Greenwich Hospital, who should be invited to meet the governors and to go very carefully over the building from top to bottom; if they decided to do nothing, and if the time arrived when it was essential to acquire another building, it would have to be sought elsewhere. Many of the men in Greenwich Hospital belonged to a class not met with in modern hospitals. The maxim of the modern hospital was to treat the largest number of cases in the fewest beds in the shortest possible time. The average duration of stay had been reduced from 35 days to 19, and meant an enormous saving in the working power of the people. There were many men in Greenwich Hospital who would not be accepted at a hospital worked on those lines, but whom, nevertheless, the Seamen's Hospital was bound to care for. If they wished to shorten the length of stay at Greenwich, there was no doubt that the James offer of a convalescent home in the Isle of Wight was a very tempting one. It seemed to him that it should be attached to the Seamen's Hospital, and it would be an excellent thing if somebody came forward to take an interest in sailors—even if only those suffering from tuberculosis were considered—and took the matter up. It was a thing that the ladies might very well do; the amount to be raised would be £1,300 a year.

A Unique Hospital.

The Seamen's Hospital was a unique hospital; it took patients regardless of colour, race, or creed; it was every sailor's friend, known to the ruling powers all over the world. As a traveller, he could speak of the gratitude felt by sailors who had been treated there. The hospital wanted more public support, and a wider area from which to draw subscriptions. There was an appeal in the very fact that the object of that hospital was the treatment of the sick of all nations, in their most helpless and friendless condition, and he thought that it should come first on the list of those who subscribed to charities, until £10,000 was added to its reliable income. Every penny was well spent, and from its great claims on Englishmen, who owed so much to sailors, the reward of giving to it in blessing and satisfaction would be very great.

Mr. Henry Wallach having spoken of the special claims of the West Coast of Africa, a member of the committee said he had known the property at Greenwich for half a century, and he thought it very well adapted for the purpose; at any rate it was very much to the sailor's fancy, more so than large modern wards would be. The establishment had all along been a very healthy one.

Thanks to the chairman, Mr. Nairne, who represents the third generation of Nairnes who have had the good of the Seamen's Hospital at heart, brought the meeting to a close.

---

### LONDON HOMŒOPATHIC HOSPITAL.

A fair attendance was secured at the annual meeting of governors of the London Homœopathic Hospital, held in the handsome board-room of the institution, under the presidency of Lord Cawdor, on Thursday, February 27th. Sir Henry Tyler, in a speech towards the close of the proceedings, which lasted about an hour, made special reference to the pleasure it gave him to see so many ladies present. Several good-humoured allusions were made by the speakers to the attitude of allopaths towards homœopathic doctors.

The Chaplain (Rev. E. C. Bedford) having opened the meeting with prayer, the secretary-superintendent, Mr. G. A. Cross, read the fifty-second annual report for 1901.

Lord Cawdor (treasurer and vice-president) moved the adoption of the report, and, referring to the expenditure from capital, said if they had this year to draw £2,000, they would have drawn the full amount of capital which they had authority to draw upon.

Mr. J. P. Stilwell, J.P., (chairman of the board), in seconding the motion, said that if this necessary expenditure—"which keeps us up to the high-water mark of efficiency"—continued to be withdrawn from capital, in five years from now they would come to an end of their available assets. Both speakers agreed that this year or next a vigorous attempt will have to be made to raise a fund of £12,000 to liquidate the amount already authorised, but the claims of the Coronation ceremonies upon so many people are deemed to render the present inauspicious to such an appeal. Appreciative hopefulness was expressed as to the results to be expected from the establishment of "The Ladies' Guild" referred to in the report, branches of which are to be shortly brought into being at Streatham, Denmark Hill, and Highgate. Allusion was also made to a "Twentieth

Century Fund" proposed by the doctors, originating from a speech by Dr. G. H. Burford at the Homœopathic Hospital, having in view the establishment of a school or the provision of other educative facilities. Personal sorrow was expressed with reference to the death of Miss J. Durning Smith, whose portrait has been added to the numerous fine pictures adorning the board-room. Dr. Richard Hughes also spoke, and the report was unanimously adopted.

Mr. Stilwell proposed a vote of sympathy and condolence with the widow and family of Mr. A. E. Chambre, an old friend and valuable coadjutor, who had died since the printing of the report, after an illness commencing last October. He had been connected with the hospital for many years, and took the greatest interest at the time of the rebuilding operations, having been Chairman of the Rebuilding Committee. Dr. Dyce Brown seconded, and the vote was carried.

The re-election of the managers and officers—including the election of Dr. Goldsbrough to replace Dr. Edwin A. Neatby, resigning—having been agreed to, as well as the usual votes of thanks, an abstract of the report of the Eastbourne Convalescent Home was read and adopted. This concluded the business.

### THE CANCER HOSPITAL.

"This has been the most interesting meeting I ever remember," was the observation of one of the governors towards the close of the proceedings at the fifty-first annual gathering of the Cancer Hospital (Free), on Wednesday, 26th ultimo, presided over by Dr. Alexander Marsden, the son of the founder of the institution. A good number of ladies were present, and the public attention now being directed toward cancer was reflected both in the committee's report and in the remarks of the various speakers.

### The Committee's Report.

The report presented by the General Committee stated that the new in-patients during the year numbered 704, and the out-patients 1,604. The total attendances numbered 16,343, being 2,705 in advance of those recorded in 1900. The average number of beds occupied was 89—1 in excess of the previous year. The ordinary income for the year was £11,910 and the legacies £2,249. The ordinary expenditure amounted to £12,393 and the extraordinary (repairs and building improvements) to £340. Since the last annual meeting the hospital had, by the death of Sir George Measom, lost the services of its chairman. For 20 years Sir George had been a member of the board, during the last 10 of which he occupied the chair. That charity, as well as many other London philanthropic institutions, had thereby been deprived of a sincere friend, a generous supporter, and a wise director. The vacancy thus caused had been filled by Dr. Alexander Marsden, son of the founder, accepting the chairmanship of the General Committee, and Lieutenant-Colonel T. R. Parr that of the House Committee. With regret the death of Mr. John Corbett, who joined the board in 1891, was also reported. During the period under review the only changes in the *personnel* of the staff were the appointment of Mr. W. Carlyle Wilson as house surgeon in place of Mr. E. Z. Davies, resigned, and of the Rev. J. Farrington Downes as chaplain.

### Chaplain's Stipend Endowment Fund.

For some time past, the committee stated, they had thought that it would be a source of comfort and blessing to many of the suffering inmates if a greater amount of spiritual attention, and more especially bedside visitation, could be procured than they felt justified in providing out of the general funds of the hospital. They therefore decided to establish a special fund for the purpose, to be called the Chaplain's Stipend Endowment Fund, the creation of which they thought would be a fitting way to commemorate the fiftieth year of the Hospital's existence. To the arrival at this decision they were most generously assisted by the Right Hon. Sir Massey Lopes, who considerably added to the many debts of gratitude the Institution already owed him by handing over a sum of £1,053 to the special fund. A special appeal for this object was issued late in the year, which had so far met with a satisfactory response. It was hoped that the amount needed, about £6,000, would shortly be forthcoming, so as to make the special fund absolutely independent of the ordinary income of the Hospital The committee were very loth to part with the services of the Rev. F. W. A. Wilkinson, M.A., who had filled the chaplain's post for some years, and had so loyally worked for the Hospital; but they thought that more personal attention was needed than could be expected from one who held such a large and important living.

Dr. Herbert Snow, the senior surgeon, in acknowledging a vote of thanks to the medical staff, said that, having been connected for 26 years with that phenomenally prosperous institution, devoted entirely to the treatment and investigation of cancer, he might fairly claim to have had as much to do with this disease as any man of his generation. He believed that the ultimate mystery of cancer was to be elucidated by investigation among the protozoa and other low forms of life which had no nervous system, and consequently felt no pain. His experience led him, years ago, to the conclusion that the lymphatic glands were actively engaged in the destruction of the diseased cells, and it was in connection with this law of nature that they must look for the discovery of a cure for cancer which would be the greatest glory of His Majesty's reign.

### Extension of the Pathological Department.

The subject of cancer had lately been very prominently before the public, and the committee were taking steps to greatly extend the Pathological Department, so as to give further facilities for the continuance of investigations into that terrible disease. Scientific research work had been carried on at that hospital since 1885, and the committee ventured to think that the gentleman who had now for some years held the position of Senior Pathologist had done at least as much as any man in the world towards the elucidation of the mysteries surrounding the origin or cause of the complaint. They felt, however, that the time had now arrived when further efforts in this direction were necessary, and they considered they were rightly interpreting the wishes of all the Governors when they expressed the opinion that the Cancer Hospital should be in the forefront of all advancements towards the solution of these questions. A scheme at present under discussion would entail an additional expenditure of not more than about £600 per annum, and it was confidently anticipated that, should the proposals be adopted, sufficient help would soon be forthcoming to enable them to be put into practice. The committee, recognising that the vast field available for study and research at this hospital should be fully utilised, thoroughly recommended co-operation in any larger scheme in conjunction with other institutions for this purpose.

Referring to this subject, the Medical Committee in their separate report, stated that the King lately commented upon the deplorable ravages of cancer, thereby directing universal attention to its origin and treatment. The committee received numerous suggestions on the latter, and, while exercising necessary discrimination, were careful not to neglect any novel methods offering prospects of benefit. It was nevertheless obvious that further research work was urgently needed. They felt most strongly that the staff of the Cancer Hospital should be the recognised pioneers in that truly scientific investigation of cancer which its increasing prevalence demands, and should be enabled fittingly to carry on the good work inaugurated by Dr. William Marsden 51 years ago. They had every reason to hope that the proposed reconstitution and extension of the pathological and research department would usher in a new era of cancer-science, and eagerly looked forward to this great step in the history of the institution. During the past year 500 surgical operations were performed, with the low mortality of 1·4 per cent.

The Chairman, in moving the adoption of the report, after a warm tribute to the character of his predecessor in the chair, Sir George Measom, whose death occurred four days after the last meeting, referred to the past year as one of the most prosperous in the history of the institution. They were nobly supported by the public, and he thought with good reason.

Colonel Parr seconded the resolution, which was carried, as were also votes of thanks to the staff, the secretary, Mr. Howell, being specially commended for the harmony prevailing, the efficiency of his department, and the increase of subscriptions due to his exertions.

The customary compliment to the Chairman brought to a close a meeting of a distinctly animated charater, as compared with the generality of such gatherings.

### ROYAL LONDON OPHTHALMIC.

### HELP URGENTLY NEEDED.

Fifteen ladies and two or three gentlemen faced the half-dozen members of the committee who supported the chairman at the annual meeting of the Royal London Ophthalmic, so long and honourably known as the Moorfields Eye Hospital, on the 27th ultimo.

Mr. H. P. Sturgis, the chairman of the committee of management, presided, excusing himself for doing so on the ground that all efforts to secure the presence of a better man had failed. The report and accounts presented, he said, were drawn up very fully so as to make the position clear. From these it appeared that 68 beds remained unopened for want of funds, 70 beds only being available for inpatients out of a total of 138, and many who ought to have been taken in having to be refused admission. It was with great pleasure that the committee were able to report that what we shall now have to get familiar with as King Edward's Fund, had made a special grant to enable 18 additional beds to be opened in 1902. The committee were naturally proud with the terms of the letter from Sir Savile Crossley, conveying the gratifying intimation, which read as follows:—

"The Prince of Wales's Hospital Fund for London.
"The Bank of England,
"December 27th, 1901.

"The Treasurer,
"Royal London Ophthalmic Hospital,
"City Road, E.C.

"Sir,—By desire of his Royal Highness, the President, I have the honour to enclose a cheque for £2,150.

"Of this sum £900 is an annual grant to open Eighteen closed beds, on the condition that by the opening of these beds Eighteen more are made available for the sick poor in your hospital; and the balance of £1,250 is a special donation for this year.

"I am also directed to inform you that your building is reported on as a very fine new building. The visitors state that all the Wards, Operating Rooms, etc., are thoroughly practical—up to date—and that your very complete Hospital requires considerable additional funds to carry on its useful work.

"Kindly acknowledge the receipt of the above.
"Yours faithfully,
"(Signed) S. CROSSLEY,
"Honorary Secretary."

Once more the burden of rates was bitterly complained of, and it was anticipated that for the current year these would amount to £1,008.

### A Little Ray of Sunshine.

Here, however, a gleam of hope shone forth, the Rev. G. H. Perry, Rector of St. Luke's, who was present, intimating that if the Holborn Board of Guardians, of which he was a member, were approached, they would probably be able to afford substantial relief in this direction, and intimating also his willingness to take charge of such an application if made—as the Chairman, of course, promptly undertook that it should be. Other items of interest referred to were that by the additional appointments to the surgical staff in 1900, the work had been considerably facilitated; that 406 applicants had been refused as unsuitable during the year by the inquiry officer, that there was reason to hope that the purchase of the old Moorfields site would soon be completed, that Lord Hillingdon had been elected treasurer, and that a special sub-committee had been appointed to revise the rules and bring them up to date. To sum up, three points were clear, the chairman said: The institution was wanted—they could fill the 50 remaining beds to-morrow if they could see their way to open them, it was efficient—its appliances were of the best and most modern, and its staff second to none, while the testimonial from King Edward's Fund spoke for itself; and lastly it was in a very serious condition from insufficient means. They would require about £12,300 for the current year's expenditure. The income they could look to with any degree of certainty was only £4,485, leaving some £8,000 still to be obtained. They could not repeat last year's loan of £5,000, and it was therefore absolutely necessary to make a special appeal. They wanted £5,169 to clear off the debt, £8,000 for the expenses of the current year, and special donations to the Rent Fund Investment to meet the ground rent of £1,210 per annum.

There was no discussion beyond Mr. Perry's remarks about the rates already mentioned, and the report and accounts, and the various votes of thanks were all duly adopted, Mr. Bland, the secretary, receiving a special meed of praise.

### THE ROYAL HOSPITAL FOR CHILDREN AND WOMEN.

### MEETING AT THE MANSION HOUSE.

A pretty and unusual incident at an annual meeting of Governors was the presentation, by a little patient, of a basket of flowers to the Lady Mayoress on Monday afternoon. Etty Dowry is a little girl of about six years old, and she was chosen to represent the patients of the Royal Hospital, who had put together their pennies to buy a royal basket of flowers—red, white and blue. Etty, who wore a white pinafore over a blue frock, and a Red-riding-hood cape cast off by *Truth's* latest doll, made her curtsey very nicely, and was thanked not only by the Lady Mayoress but by the Lord Mayor himself. It was certainly a red-letter day for her. There were about fifty Governors present, and with the Lord Mayor, who was ushered in in state, was Sir Edwin Durning Lawrence, chairman, and Mr. Consibee, the secretary and superintendent of the hospital. The committee had seats near the table, and the rest of the company faced the speakers. The sheriffs, who had been expected to attend with their ladies, were unfortunately obliged to be elsewhere, and their absence was apologised for by the Lord Mayor. Among other supporters who were unavoidably absent were His Grace the Duke of Norfolk, the Lord Bishop of Rochester and Lady Emma Talbot, and Mr. Labouchere.

Mr. Consibee read an abstract of the report for 1901; and the Lord Mayor, in moving the adoption of the report and the re-election of the treasurer, chairman, and other officers, alluded to the founding of the Royal Hospital in 1816.

The Lord Mayor's Appeal.

The hospital was founded, he said, in the Mansion House, under the auspices of the Lord Mayor of that time, the Duke of Kent and the Duke of Sussex being present. At that time it was a hospital quite up to date, but an institution of this kind, situated in a very poor district of London, ministering to the needs of a portion of humanity which must always command sympathy and charity—namely, children and women—must keep pace with the demands made upon it, and it had become absolutely necessary to extend it and bring it up to date. When the work of the past year was considered, it became obvious that the hospital was supplying real needs: there were 488 in-patients, and 8,044 out-patients, who made 41,245 attendances; there were 416 operations, and the number of deaths, the majority being those of young children brought in too late, was only 68. Practically the beds were full all the year round. The average cost of each in-patient was seven guineas and fourpence, and that of each bed or cot occupied £78, which seemed to him very moderate. The average cost of each out-patient was 2s. 2d. These, his lordship continued, were statistics that spoke for themselves; they would appeal to all charitably minded people. This work could not, however, be carried on without help from the public. The Management Committee, moreover, were about to begin the work of re-building, in the full assurance that the charitable public, who were never appealed to in vain, would relieve them from the financial anxiety in which they were at present placed. It was for this re-building that he appealed. He believed that not only would those who were present do their best, but that they would interest their friends, so that the necessary funds would be forthcoming to make this hospital thoroughly abreast of the times. He would not directly appeal to his friends of the press to advocate its claims, because over and over again they had proved themselves ready to do all in their power to help, and he knew they would not fail now. He appealed to the charitable public to come to the help of the children and women of South London and provide the necessary means for the rebuilding of the hospital on its present site.

The Hospital to be Rebuilt.

The Treasurer (Mr. J. Topham Richardson) said this was the most remarkable meeting in the history of the Royal Hospital, which was about to take the responsible step of rebuilding. It had been shown by the Lord Mayor how much the hospital was needed, and it was demonstrable that this was the right time in which to build. He had been asked whether he did not feel the responsibility as a very great one, and he had replied that it was less now than it was twelve months ago, when the decision had not been made. Now the committee had put its shoulder to the wheel, and £10,000 must be found in the next few months. Altogether the sum required would be £25,000, to make the building complete; towards that sum about £9,000 was in hand.

Sir Edward Durning Lawrence, chairman, agreed that the meeting was a record one in the history of the hospital. The Stamford Street premises would not be rebuilt immediately, as had been intended, owing to the difficulty of acquiring certain premises without paying a prohibitory price for the short leasehold interests. The site was therefore being adapted for temporary accommodation, both for in- and out-patients, and it was hoped that this would be completed by next May, when the old hospital in Waterloo Bridge Road would be pulled down, and rebuilt with all possible speed. This was the oldest children's hospital in London, but because people went by cab to Waterloo Station it was overlooked.

The following resolution was passed:—

"That the Royal Hospital for Children and Women is in every way worthy of support, and has strong and peculiar claims upon the City of London; and that a subscription list be at once opened to make up the annual deficit, and to complete the sum required for the rebuilding and extension now commenced."

Dr. Gow drew attention to the crowded and unhealthy state of the out-patients' department when it was full; he had an affection for the old place, but the saying about the value of old wines and old books could not be made to apply to old hospitals; times changed, and it was necessary to move with them. He hoped the new building would represent the highest skill, and though the results had been good in the old hospital, they would be better still in a more modern one. This meeting was unique, he thought, in having a patient present, and one who had benefited greatly by the treatment in every way.

The Lord Mayor having announced that the subscription list was open, the following sums were acknowledged:—£30 9s. in new annual subscriptions, and £102 0s. 6d. in donations. A very warm vote of thanks to the Lord Mayor and the Lady Mayoress concluded the meeting.

The new hospital will contain 200 beds instead of 54; isolated wards, which will obviate the closing of the hospital for cleaning; excellent theatres and commodious out-patient departments, with separate consulting and waiting-rooms for each member of the medical and surgical staff. There will be ample corridors on each front for the open-air treatment of patients, and these will also afford permanent means of egress from each ward in case of fire. The site is a corner one, facing Waterloo Bridge Road and Stamford Street. Messrs. Waring and Nicholson are the architects.

### ST. JOHN'S HOSPITAL FOR DISEASES OF THE SKIN.

No Quorum again for the Special Meeting.

The question of the non-attendance of governors at hospital meetings has reached an acute stage in connection with St. John's Hospital for Diseases of the Skin. By the rules of the institution a quorum of 25 is requisite for the holding of the annual general meeting and 40 for a special meeting, and for the past two years at least considerable trouble has been experienced in getting a meeting at all. It is desired to alter the rules so as to constitute a smaller number a legal quorum, but the difficulty seems to be to get the necessary 40 together to sanction this.

On the 28th ult. a special general board of governors was advertised for 4.30 at the Westminster Palace Hotel, to be followed by the annual general meeting. The special meeting was for the purpose of granting permission to the Board of Management to rebuild the hospital premises on the site of the existing buildings, Leicester Square, in the event of their being able to obtain favourable terms for so doing; and also to alter the rules by substituting 30 for 40 as the quorum. At 4.45, however, only 25 governors were present and the annual meeting was therefore proceeded with.

In the absence of Lord Chesterfield (the President), who was detained in Paris on legal business, Mr. C. Guy Pym took the chair. He thanked those ladies and gentlemen who had taken the trouble to be there for their presence, remarking that never before when he had been there had they got the proper number, though on one occasion they waited more than an hour, and were even then unable to transact their business. He was glad that was not altogether the case that day, though there seemed no likelihood of the requisite 40 for the special meeting turning up. It was clear that they must devise some means to reduce the quorum Last year three meetings had to be called, and they would notice an item in the balance-sheet of £56 for annual meeting expenses, which certainly ought not to be there.

The adoption of the report having been seconded, Dr. Dennis Vinrace suggested that they might, under the Act of 1875, obtain relief from the Registrar of Friendly Societies with regard to their quorum. Mr. Van Praagh, the honorary solicitor, however, said he thought Dr. Vinrace was better acquainted with his own profession than with the law. There was another Act since that of 1875, and the Registrar could not help them. However, they were preparing to take special steps in the matter.

Mr. A. F. Joiner asked how it was that nothing had been received from King Edward's Fund? The Chairman said application had been duly made and a letter which merely stated that the Council were unable to make a grant. Dr. Vinrace was proceeding to ask if the reason for this was known, but the Chairman said they could not go into that, they must get on with the business. He therefore put the resolution that the report be adopted, and this was carried *nem. con.*

On the motion to re-elect the honorary officers and board of management Mr. Joiner proposed to add the whole of the medical officers. Dr. Morgan Dockrell, however, pointed out that there was a medical committee who delegated one of their number to the board, and said that at one time they all had seats, but it became an irksome tie and they asked to be relieved, and now served each in turn. On being pressed only two hands were held up for the amendment, which was therefore lost.

Votes of thanks concluded the proceedings.

### NATIONAL ORTHOPÆDIC HOSPITAL.

The annual meeting of the above hospital was held on February 28th, His Grace the Duke of Marlborough (the President) in the chair.

The Chairman, in moving the adoption of the report of the committee and the statement of accounts, said the finances of the hospital were in a satisfactory condition. The receipts last year were £2,528, being £300 in excess of those of the year 1900. It was to be regretted that the hospital had not more annual subscribers, and he hoped that efforts would be made to increase the number. During the last year there had not been one bed vacant in the hospital, while an average of 60 cases accepted by the surgeons as in-patients had been waiting to come in all the year round. This conclusively showed the great need that existed for increased accommodation for children who were suffering from the deformities with which the Orthopædic Hospital had to deal. It was remarkable that with so small a building they had been able to render aid to so many people. They would see by the report that the committee had sanctioned a new department of work in connection with the children and young patients in the hospital. They had arranged to have instruction given them in kindergarten and other educational work. He fully approved of this arrangement, and believed it would receive the sanction of their supporters.

The Rev. T. Stephen seconded the motion for the adoption of the report, which was carried.

On the motion of the Rev. G. P. M'Kay, thanks were voted to the committee, and the retiring members of that body were re-elected.

*NOW READY.*

**BURDETT'S HOSPITALS & CHARITIES, 1902.**

**The Year-book of Philanthropy and Hospital Annual.**

**By Sir HENRY BURDETT.**

Thirteenth Year. Crown 8vo., over 1,000 pp.
Cloth, **5/-** post free.

THE SCIENTIFIC PRESS, LIM., 28 & 29 Southampton Street, Strand, London, W.C.

**Tender.**

**SEAMEN'S HOSPITAL SOCIETY.**

